# Skimmianine attenuates liver ischemia/reperfusion injury by regulating PI3K–AKT signaling pathway-mediated inflammation, apoptosis and oxidative stress

**DOI:** 10.1038/s41598-023-45354-2

**Published:** 2023-10-25

**Authors:** Cheng-long Huo, Bing Wang, Xuewen Zhang, Zhen-Gang Sun

**Affiliations:** Department of Hepatobiliary Surgery, Jingzhou Hospital Affiliated to Yangtze University, No. 26, Chuyuan Avenue, Jingzhou District, Jingzhou, Hubei China

**Keywords:** Hepatocytes, Apoptosis

## Abstract

Liver ischemia/reperfusion (I/R) injury is a common injury after liver transplantation and hepatectomy. Skimmianine (Ski) has antibacterial, antiviral pharmacological effects. However, it is not clear whether Ski has a protective effect against liver I/R injury. In the present study, we established a mouse liver I/R model and an AML12 cell hypoxia-reoxygenation (H/R) model, both pretreated with different concentrations of Ski. Serum transaminase levels, necrotic liver area, cell viability, inflammatory factors, oxidative stress and apoptosis-related levels were measured to assess the protective effect of Ski against liver I/R injury. Western blotting was used to detect apoptosis-related proteins and PI3K–AKT pathway-related proteins. Mice and cells were also treated with PI3K inhibitor LY294002 to assess changes in indicators of liver injury. The results showed that Ski significantly reduced transaminase levels, liver necrosis area, oxidative stress, and apoptosis levels in mice with I/R. Ski also inhibited cell injury and apoptosis after H/R. Moreover, Ski activated phosphorylation of PI3K–AKT pathway-related proteins after liver I/R and cell H/R. Importantly, the PI3K inhibitor LY294002 effectively reversed the alleviation of I/R injury caused by Ski. These results confirm that Ski exerts a protective effect against liver I/R injury through activation of the PI3K–AKT pathway.

## Introduction

Liver ischemia/reperfusion (I/R) injury is a pathophysiological process in which liver damage is further exacerbated after a period of ischemia in liver tissue followed by reperfusion of blood flow. Liver I/R injury commonly occurs after liver transplantation, hepatectomy and severe hemorrhagic shock^1,2^. The mechanisms of liver I/R injury are complex, which include inflammatory damage, oxidation response, apoptosis, and endoplasmic reticulum stress as possible mechanisms of action^3^. During I/R injury, activation of Kupffer cells, macrophages, and mitochondrial damage promote reactive oxygen species (ROS) production and inflammatory responses, leading to liver tissue damage. A vicious cycle of ROS and oxidative stress, as well as mitochondrial dysfunction, contribute to the exacerbation of I/R injury^4,5^. Apoptosis is a form of programmed cell death, and apoptosis also plays an important role in liver I/R injury^6^. Currently, there is a lack of very effective treatment modalities for liver I/R injury^2^. Therefore, it is clinically important to explore a new and effective treatment to reduce liver I/R injury. The discovery of an effective drug that can reduce I/R injury in the liver could greatly contribute to the advancement of hepatobiliary surgery^7,8^.

Phosphatidylinositol-3-kinase (PI3K) is an important molecule in the regulation of cell growth, metabolism and apoptosis. Protein kinase B (AKT), a serine-threonine kinase, is a kinase activated by phosphorylation. AKT is regulated by PI3K and exerts various effects on cellular processes including modulation of cellular inflammation, ROS, proliferation and apoptosis^9,10^. It has been shown that activation of AKT attenuates the cellular inflammatory response and reduces apoptosis^11,12^. Many studies have also demonstrated that activated AKT can attenuate I/R injury in the heart, kidney, brain, and liver^10,13–15^.

Skimmianine is a natural furoquinoline alkaloid that can be obtained from plants of belonging to the Rutaceae family^16^. Some studies have shown that Ski has antibacterial, antiviral, pharmacological effects by inhibiting acetylcholinesterase and nitric oxide (NO) production^17–19^. In addition, Ski has also been reported to have potent anti-inflammatory effects. Its mechanism of action includes inhibition of TNF-α and IL-6 gene transcription, as well as inhibition of NO, prostaglandin E2 and superoxide anion production^16,20^.

However, no studies have been reported on whether Ski can attenuate liver I/R injury. The further anti-inflammatory and antioxidant mechanisms of Ski also need to be further elucidated. In the present study, our group aimed to investigate the role of Ski on liver I/R injury and further mechanisms.

## Materials and methods

### Animals

Male C57BL/6J mice (6–8 weeks) were obtained from the Hubei Provincial Laboratory Animal Public Service Center (Wuhan, China). All animals were housed in a specific pathogen-free environment, had free access to food and water, and had a 12-h dark/light cycle. Mice were cared for according to NIH guidelines for laboratory animals. The study was carried out in compliance with the ARRIVE guidelines. Animal experiments were approved by the Ethics Committee of the Jingzhou Central Hospital.

### Drug treatment and liver I/R injury model

Skimmianine (HY-N2081, MedChemExpress, Shanghai, China) was dissolved in 0.5% carboxymethyl cellulose sodium aqueous solution to different concentrations. Mice were randomly divided into sham-operated group, Ski group, I/R group and I/R + Ski group. The experiment was started after 14 days of continuous Ski gavage (once a day) prior to modeling, and the control group was given the same amount of dissolving solvent gavage. To further investigate the protective mechanism of Ski against liver I/R injury, PI3K inhibitor LY294002^21^ (20 mg/kg) was injected intraperitoneally 1 h prior to model in I/R + Ski + LY294002 group.

In this study, a 70% liver I/R injury model was established using mice^22^. Briefly, mice were anesthetized with an intraperitoneal injection of 1% sodium pentobarbital. After a median abdominal incision, the individual lobes of the liver were separated under the microscope and the vessels and bile ducts of the left and middle lobes of the liver were clamped using microvascular clips. After 1 h, the vascular clips were removed and reperfusion was initiated. The procedure in the sham-operated group was the same as that in the model group, but without vascular clamping of the liver. After 6 h of reperfusion, the mice were euthanized via cervical dislocation, and the absence of fluctuations in the thoracic cavity, respiration, and heart rate confirmed mortality. Then the tissues and blood specimens from the mice were obtained for further experiments.

### Alanine aminotransferase (ALT) and aspartate aminotransferase (AST) assessments

After liver reperfusion, blood samples were collected from the orbit and left at room temperature for 30 min^23^. Subsequently, serum was obtained by centrifuging the blood samples at 3500 g for 5 min. The obtained serum was used for the detection of serum levels of ALT and AST using the relevant kits (BC1555 and BC1565, Solarbio, Beijing, China).

### Hematoxylin and eosin (HE) staining

Liver tissues were fixed in 10% formalin, embedded in paraffin, cut into 5 μm thick paraffin sections. HE staining was performed according to the instructions for HE staining reagents (servicebio, Wuhan, China), observed under an inverted light microscope and photographed (Olympus, Japan)^24^. The area of liver necrosis was analyzed by Image J.

### Terminal deoxynucleotidyl transferase-mediated nick end labeling (TUNEL)

Five μm Paraffin-embedded liver tissue sections were dewaxed, and each section was soaked by treating with 100 μL proteinase K dropwise for 20 min at 37 °C in a wet box. After rinsing three times with PBS for 5 min each, TUNEL reaction solution was added dropwise and rinsed three times with PBS for 5 min each in a light-proof wet box at 37 °C^25^. The nuclear were stained by DAPI. Sections were then sealed with an anti-fluorescence quencher and photographed under a fluorescent microscope (Olympus, Japan). The total number of cells and the number of positive cells (nuclei in red) were counted using Image J analysis software.

### Cell culture and H/R model

AML12 cells were purchased from Procell Life Science & Technology Co., Ltd. (Wuhan, China) and cultured in Dulbecco's modified Eagle's medium/nutrient mixture F-12 medium (Procell, Wuhan, China) in a cell incubator with 5% CO_2_ at 37 °C. AML12 cells from the H/R group were incubated in serum-free medium in a triple-gas incubator (5% CO_2_, 94% N_2_, 1% O_2_) for 12 h to complete this step of hypoxia. Immediately after the completion of hypoxia, the serum-free medium was replaced with normal medium containing 10% fetal bovine serum (FBS) for reoxygenation, and the cells were placed in the normal incubator for 6 h to complete the reoxygenation^26^.

### Oxidative stress analysis

Mouse liver tissue or AML12 cells were homogenized with PBS or extract and centrifuged at 8000 g for 10 min. The supernatant was collected, and the protein concentration was quantified using the BCA Protein Concentration Assay Kit^27^ (PC0020, Solarbio, Beijing, China). The malondialdehyde (MDA) level, Superoxide Dismutase (SOD), and Glutathione (GSH-Px) activities were detected according to the kit instructions (BC0025, BC0175 and BC1195, Solarbio, Beijing, China). Reactive oxygen species (ROS) generation in liver tissue was detected using dihydroethidium (DHE) staining (GDP1018, Servicebio, Wuhan, China). Briefly, fresh frozen liver tissue was cut into 10 μm sections and incubated with DHE (10 µM/L) at 37 °C for 30 min in the dark, and images were captured using a fluorescence microscope (Olympus, Japan)^28^.

### Cell counting kit-8 assay

AML12 cells were inoculated in 96-well plates at 5 × 10^3^ cells per well^29^, and the cells were treated with Ski or H/R. After 24 h of Ski treatment or after H/R treatment, 10 μl of CCK-8 reagent (C0037, Beyotime, Shanghai, China) solution was added to each well of the plate and incubated for 1 h at 37 °C and 5% CO_2_, the absorbance was measured by a micro-plate reader (Bio-Rad, USA) at 450 nm^30^. The relative proliferation ratio was calculated by as follows: (experimental absorbance value/control absorbance value) × 100%^31^. At least three independent tests were performed, each independent test included six wells.

### Flow cytometry analysis

After reoxygenation, the culture medium was discarded. After washing with PBS once, the cells were digested using trypsin without EDTA to collect the cells, centrifuged at 200 g for 5 min, and 100 μL suspension was added to resuspend the cells and stained with 5 μL Anexin V-FITC and 5 μL PI staining solution (CA1020, Solarbio, Beijing, China), which the cells were incubated in at room temperature for 15 min in the dark, and then detected by flow cytometry (BD Biosciences, USA)^32^.

### Real-time polymerase chain reaction (PCR) analyses

Total RNA was isolated using Trizol reagent (Solarbio, Ltd, Beijing, China) according to the manufacturer's instructions, and 1 μg of RNA per sample was reverse transcribed using the PrimerScript Reverse Transcription Kit (R323-01, Vazyme, Nanjing, China) according to the manufacturer's protocol. After reverse transcription, PCR reactions were performed using the SYBR qPCR Master (Mix reagent (Q711-02, Vazyme, Nanjing, China) kit. GAPDH was used as an internal reference, and the expression level of the target gene was calculated using the 2^−△△Ct^^33^. The primers used in the experiments are listed in Table 1.

**Table 1:** Gene-specific quantitative-polymerase chain reaction primers.

Primer	Primer sequence
IL-1β F	5′-TGCCACCTTTTGACAGTGATG-3′
IL-1β R	5′-TGATGTGCTGCTGCGAGATT -3′
IL-6 F	5′-TGATGGATGCTACCAAACTGGA-3′
IL-6 R	5′-TGTGACTCCAGCTTATCTCTTGG-3′
TNF-α F	5′-CCCTCACACTCACAAACCAC-3′
TNF-α R	5′-ACAAGGTACAACCCATCGGC-3′
CXCL10 F	5′-TGCCGTCATTTTCTGCCTCA-3′
CXCL10 R	5′-AGGCTCGCAGGGATGATTTC-3′
GAPDH F	5′-GGAGAGTGTTTCCTCGTCCC-3′
GAPDH R	5′-ATGAAGGGGTCGTTGATGGC-3′

### Western blotting

Total proteins were extracted using RIPA lysis buffer supplemented with a mixture of PMSF, peptidase and phosphatase inhibitors. Protein concentrations were measured using a BCA Protein Concentration Assay Kit (PC0020, Solarbio, Beijing, China). Proteins were separated using SDS-PAGE on 10% or 12% SDS gels (30 μg per lane), transferred to PVDF membranes and incubated with primary antibodies at 4 °C overnight, followed by incubation with appropriate horseradish peroxidase (HRP)-coupled secondary antibodies for 1 h at room temperature. BeyoECL Plus (SA00001-1, Beyotime, Shanghai, China) was used for signal visualization. Images were collected using a Fushon Fx (Vilber Lourmat) imaging system (Marne-la-Vallée, France) (Original protein results are provided in Supplementary file).

### Molecular docking

The two-dimensional structure of Skimmianine was drawn using Chemdraw (version 19.0; Cambridge soft corporation) and subsequently imported into Chemdraw 3D (version 15.0; Cambridge soft corporation) for energy minimization using the MM2 module to obtain the lowest energy advantage concept and saved as a mol 2 file. The PI3K protein structure (AF-Q3U6Q4) was downloaded from AlphaFold Protein Structure Database and subsequently visualized separately using PyMOL (The PyMOL Molecular Graphics System, version 2.0.6; Schrödinger, LLC), and then the ligand and receptor were saved separately as PDPQT using Mgtools 1.5.6^34^ after processing by dehydration, hydrogen addition, charge calculation, and merging of non-polar hydrogen, etc. The docking of ligand and receptor was performed using Autodock vina 1.1.2^35^ and visualized by PyMOL.

### Statistical analysis

SPSS software (version 22.0; IBM Corp.) was used for statistical analyses. A one-way ANOVA followed by a post hoc Bonferroni correction was used for the statistical analysis more than two groups. A Student’s t-test was used to compare the differences between two groups. *P* < 0.05 was considered to indicate a statistically significant difference.

## Results

### Skimmianine attenuates liver I/R injury in mice

**Figure 1:**
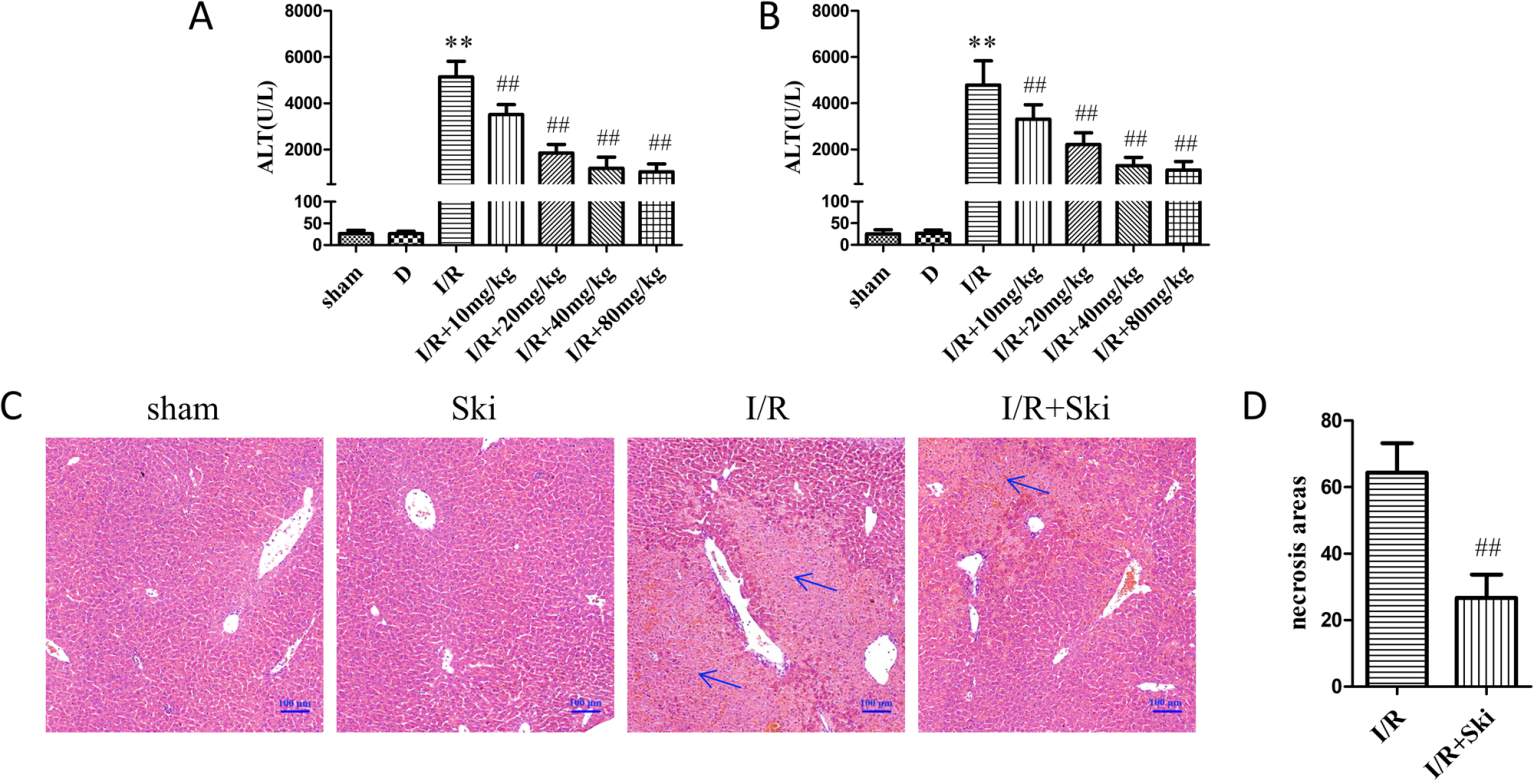
Skimmianine attenuates liver I/R injury in mice. (**A** and **B**) Serum ALS and AST contents of mice in different treatment groups. (**C**) Representative H&E staining and (**D**) necrotic area statistical analysis of liver tissue. ^**^P < 0.01 versus sham group; ^##^*P* < 0.01 versus I/R group.

We treated different groups of mice with different concentrations of Ski and subsequently constructed a liver I/R injury model. The results showed that the levels of ALT and AST were significantly (*P* < 0.01) reduced in Ski treated mice, and the protective effect was most obvious in the 40 mg/kg group (Fig. 1A,B). Meanwhile, we performed HE staining on liver tissues of mice and counted the necrotic area. The results showed that the necrotic area of Ski -treated mice was significantly (*P* < 0.01) reduced compared to the I/R group (Fig. 1C,D). Therefore, in the subsequent experiments, we selected 40 mg/kg concentration of Ski for treatment. These results suggest that Ski could significantly reduce liver I/R injury.

### Skimmianine alleviates inflammation response in mouse liver I/R injury

**Figure 2:**
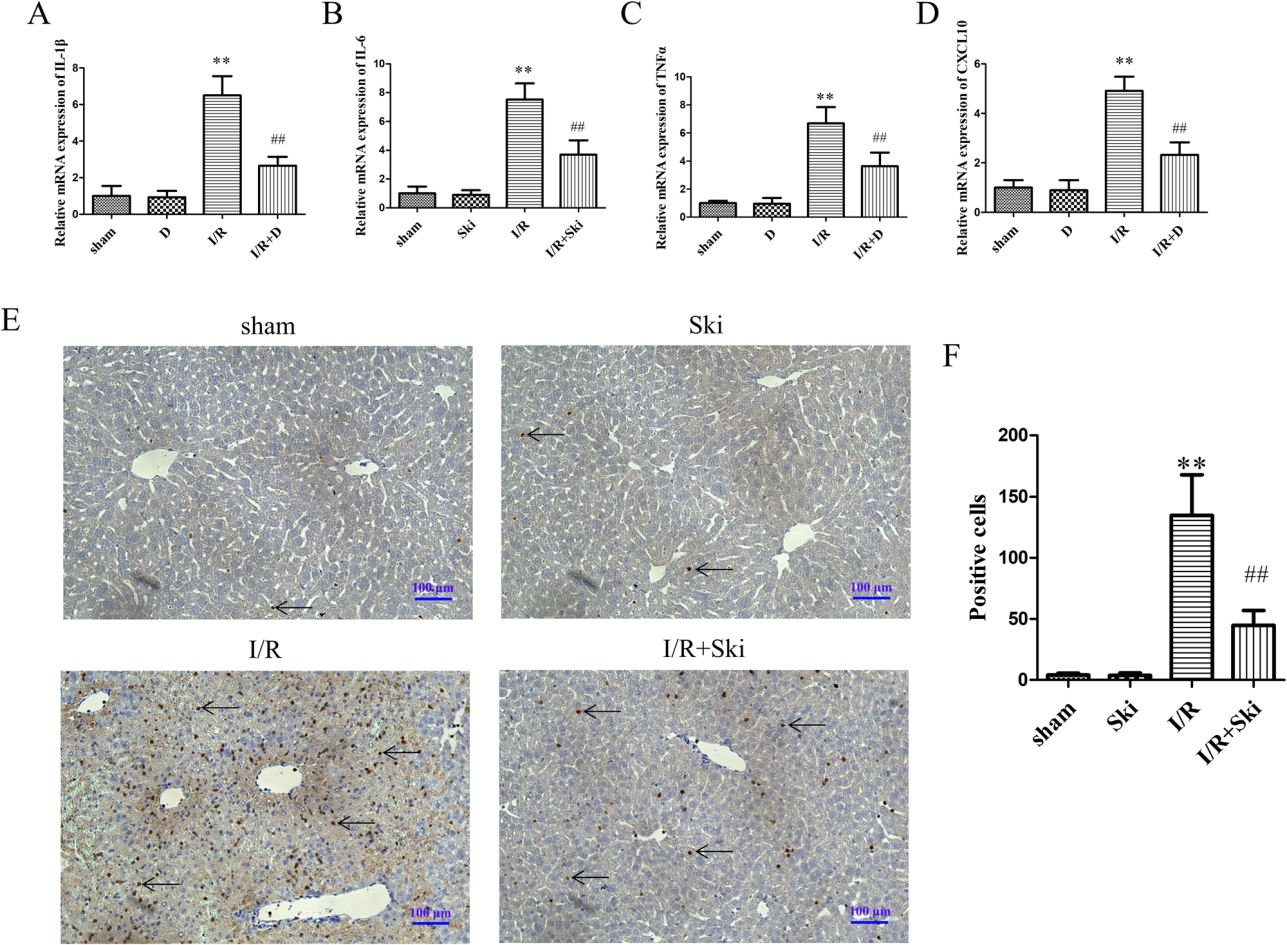
Skimmianine alleviates inflammation in mouse liver I/R injury. (**A**–**D**) The mRNA expression of inflammatory cytokines IL-1β, IL-6, TNF-α, and CXCL10 of mice in different treatment groups. (**E**) Representative MPO immunohistochemical staining of liver tissues and (**F**) statistical analysis from different groups. ^**^P < 0.01 versus sham group; ^##^*P* < 0.01 versus I/R group.

We used PCR technique to detect the changes of IL1-β, IL-6, TNFα, and CXCL10 in mouse liver tissues. The results showed that Ski treatment could significantly (*P* < 0.01) attenuate IL1-β, IL-6, TNFα, CXCL10 levels after I/R compared with the I/R group alone (Fig. 2A–D). Meanwhile, we performed myeloperoxidase (MPO) staining of liver tissues from mice using immunohistochemistry, and the results also showed that Ski treatment significantly (*P* < 0.01) attenuated MPO expression after I/R (Fig. 2E,F). These results suggest that Ski reduced the inflammatory response in liver I/R injury.

### Skimmianine alleviates oxidative stress after liver I/R in mice

**Figure 3:**
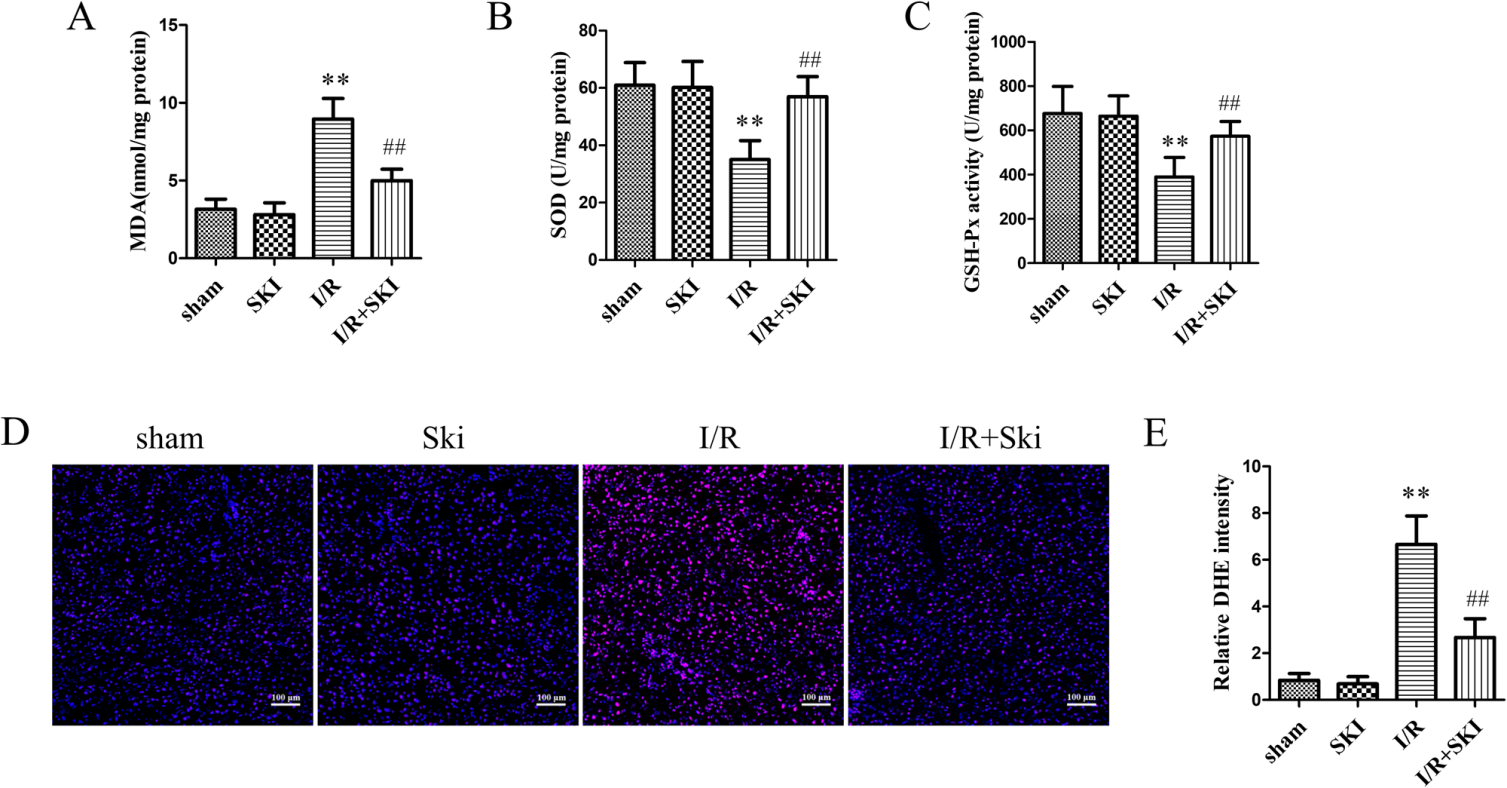
Skimmianine alleviates oxidative stress after liver I/R in mice. (**A**) MDA content, (**B**) SOD activity and (**C**) GSH-Px activity in liver tissues of mice in different treatment groups (**D**) Representative DHE staining and (**E**) fluorescence intensity statistical analysis of liver tissues in different treatment mice. ^**^P < 0.01 versus sham group; ^#^*P* < 0.05 and ^##^*P* < 0.01 versus I/R group.

To detect the level of oxidative stress in mice after liver I/R, we measured the levels of SOD, GSH-PX and MDA in the liver tissues. The results showed that Ski treatment signifcantly (*P* < 0.01) reduced the elevated MDA levels induced by I/R, while increased SOD and GSH-Px activity caused by I/R compared with the I/R group (Fig. 3A–C). Meanwhile, we performed DHE staining to detect ROS content in mouse liver tissues, and the results confirmed that Ski could significantly (*P* < 0.01) reduce the ROS content after I/R injury (Fig. 3D,E). These results suggested that Ski can reduce oxidative stress in liver I/R injury.

### Skimmianine alleviates apoptosis in mouse liver after I/R

**Figure 4:**
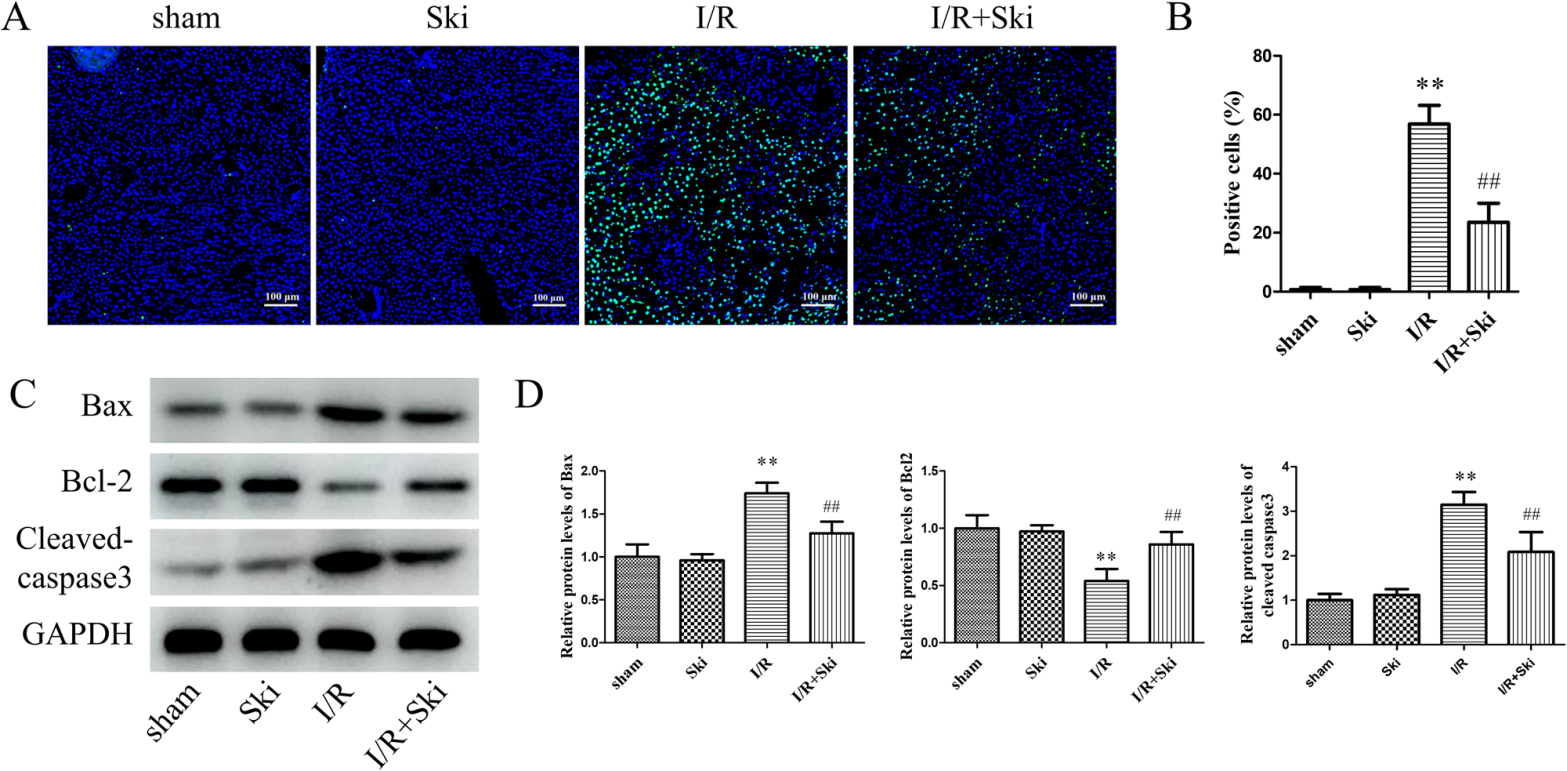
Skimmianine alleviates apoptosis in mouse liver after I/R. (**A**, **B**) TUNEL staining and statistical analysis of liver tissue in different treatment mice. (**C**) The protein expression of Bax, Bcl-2 and Cleaved-caspase 3 and (**D**) protein quantitative analysis of liver tissue in different treatment mice. ^**^P < 0.01 versus sham group; ^##^*P* < 0.01 versus I/R group.

To further investigate whether Ski can affect the level of apoptosis after liver I/R injury. We performed TUNEL staining on mouse liver tissue, and the results showed that Ski treatment significantly (*P* < 0.01) reduced the percentage of TUNEL positive cells compared with the I/R group alone (Fig. 4A,B). Meanwhile, we detected apoptosis-related proteins in mouse livers. The results showed that Ski treatment could significantly (*P* < 0.01) reduce the protein expression levels of Bax and Cleaved-caspase3 after liver I/R injury. Correspondingly, Ski significantly (*P* < 0.01) elevated the protein expression level of Bcl-2 in liver after I/R injury (Fig. 4C,D). For mice without I/R treatment, Ski did not exert a significant effect. These results suggest that Ski alleviated the elevated apoptosis caused by I/R injury.

### Skimmianine alleviates H/R-induced cell injury

**Figure 5:**
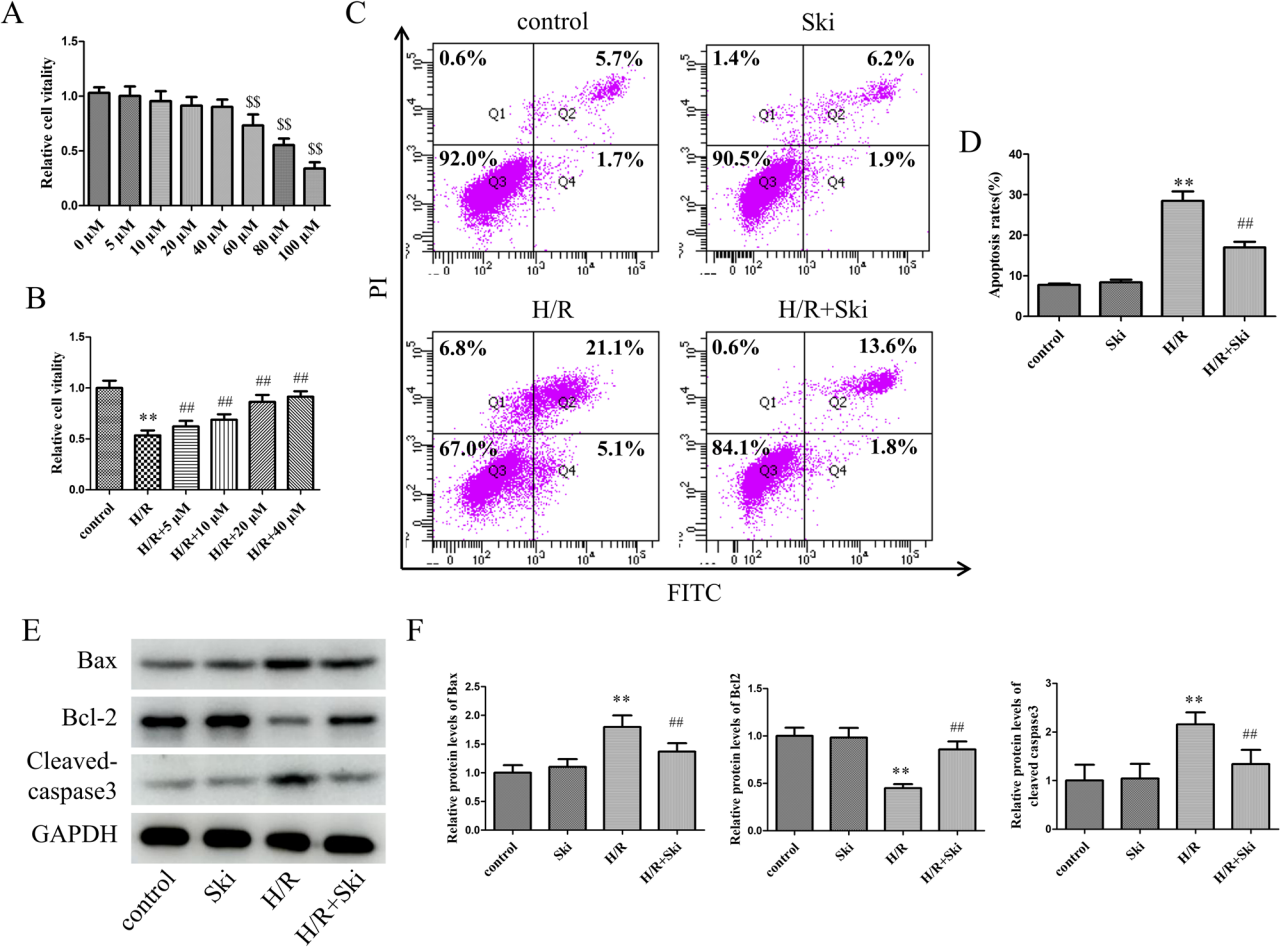
Skimmianine alleviates H/R-induced cell injury. (**A**) Effect of different concentrations of Ski on the activity of AML12 cells under normoxia. (**B**) Effects of different concentrations of Ski on the activity of AML12 cells under H/R. (**C** and **D**) Apoptosis rate detection and statistical analysis by flow cytometry. (**E**) The protein expression of Bax, Bcl-2 and Cleaved-caspase 3 and (**F**) protein quantitative analysis in different treatment cells. ^$$^*P* < 0.01 versus 0 μM group; ^**^P < 0.01 versus control group; ^##^*P* < 0.01 versus H/R group.

We first treated the AML12 cell line with different concentrations of Ski and determined the cell viability by CCK-8 assay after 24 h of treatment. The results showed that Ski concentrations of 40 μM and below had no significant (*P* > 0.05) damaging effect on the AML12 cell line (Fig. 5A). Therefore, in the next H/R cell treatment, we used Ski concentrations below 40 μM for cell treatment. By performing CCK-8 assay on the cells after H/R, we found that Ski at 40 μM provided the most significantly (*P* < 0.01) protection to the cells after H/R (Fig. 5B). Therefore, subsequent cell assays were performed using 40 μM Ski for treatment. We measured the percentage of apoptosis in cells after H/R using flow cytometry and demonstrated that Ski significantly (*P* < 0.01) reduced the increase in apoptosis due to H/R (Fig. 5C,D). Correspondingly, we measured apoptosis-related proteins. The results showed that Ski significantly (*P* < 0.01) reduced the protein expression of Bax and Cleaved-caspase3 compared to the H/R group, while Ski significantly (*P* < 0.01) enhanced the protein expression of Bcl-2 (Fig. 5E,F). These results suggest that Ski could attenuate the cell damage caused by H/R and reduce apoptosis after H/R.

### Skimmianine activates PI3K–AKT pathway after liver I/R

**Figure 6:**
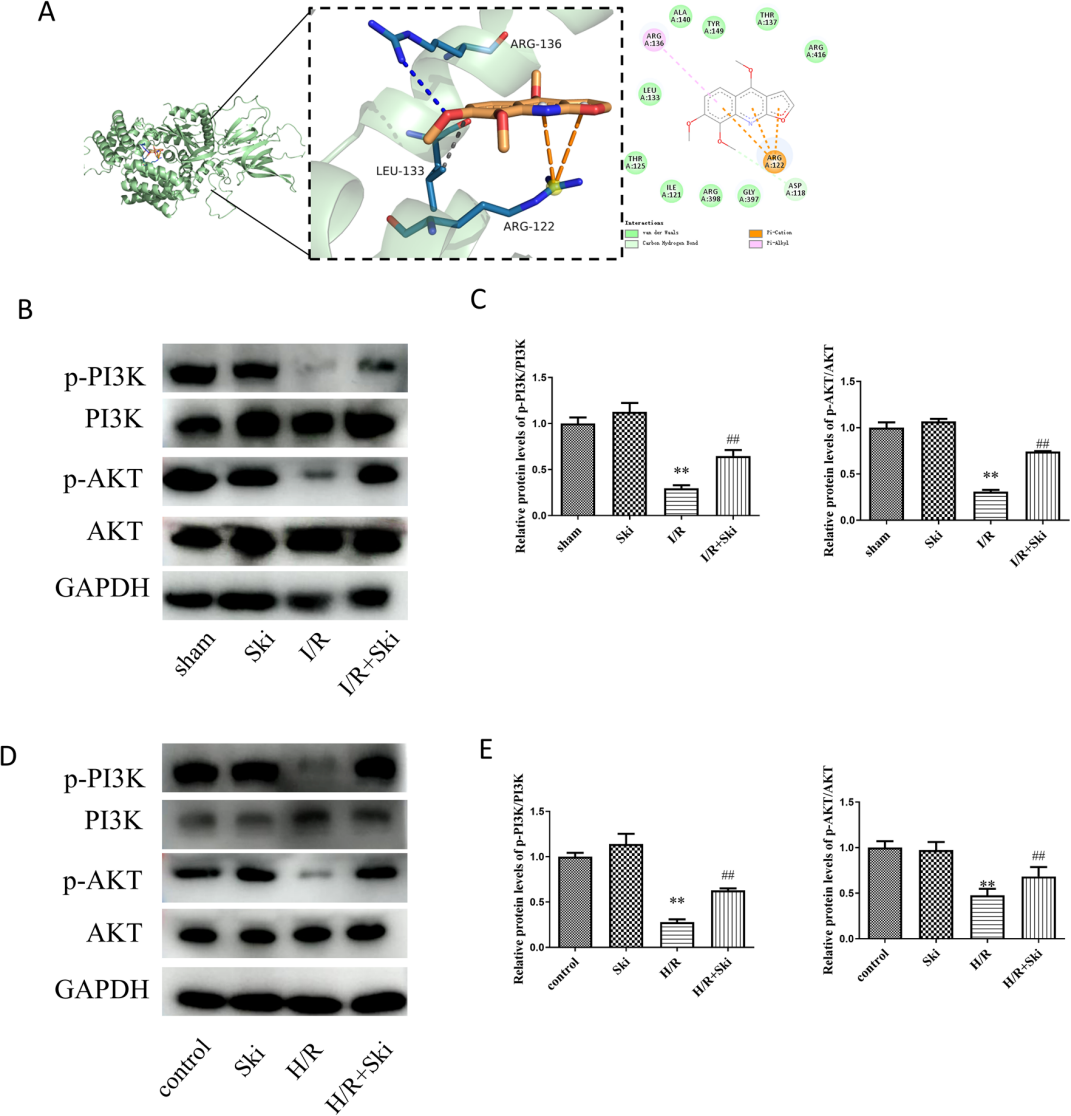
Skimmianine activates PI3K–AKT pathway after liver I/R. (**A**) The docking results of Ski and PI3k molecules. (**B**) The protein expression of p-PI3K, PI3K, p-AKT, AKT and (**C**) protein quantitative analysis in different treatment mice. (**D**) The protein expression of p-PI3K, PI3K, p-AKT, AKT and (**E**) protein quantitative analysis in different treatment cells. ^**^P < 0.01 versus sham group; ^##^*P* < 0.01 versus I/R group; ^$$^*P* < 0.01 versus control group; ^&&^*P* < 0.01 versus H/R group.

The PI3K/AKT signaling pathway plays a crucial role in regulating inflammation, oxidative stress, and apoptosis following liver ischemia–reperfusion injury. Previous studies have indicated a potential link between Skimmianine and the PI3K pathway, suggesting that Skimmianine may be associated with the modulation of this pathway^36^ We docked PI3K with Ski using molecular docking. The results revealed that Ski was able to bind effectively to the active pocket of PI3K with a binding energy of − 6.3 kcal/mol, indicating that Ski is able to bind PI3K more spontaneously (Fig. 6A)^37^. Furthermore, we examined PI3K–AKT pathway proteins in mouse liver tissues after I/R injury. Our results showed that Ski could significantly (*P* < 0.01) activate the expression of phosphorylated PI3K (p-PI3K) as well as phosphorylated AKT (p-AKT) in mice after I/R injury, but the expression of total PI3K as well as AKT proteins did not change significantly (*P* > 0.05) (Fig. 6B,C). Meanwhile, we also obtained the same results in the H/R model of AML12 cells. Ski significantly (*P* < 0.01) activate the expression of p-PI3K as well as p-AKT in AML12 cells after H/R, but the expression of total PI3K as well as AKT protein was not significantly (*P* > 0.05) changed (Fig. 6D,E). These evidences suggest that Ski could activate the inbitation of PI3K–AKT pathway in mouse liver I/R injury.

### Skimmianine inhibits liver I/R injury via PI3k–AKT pathway

**Figure 7:**
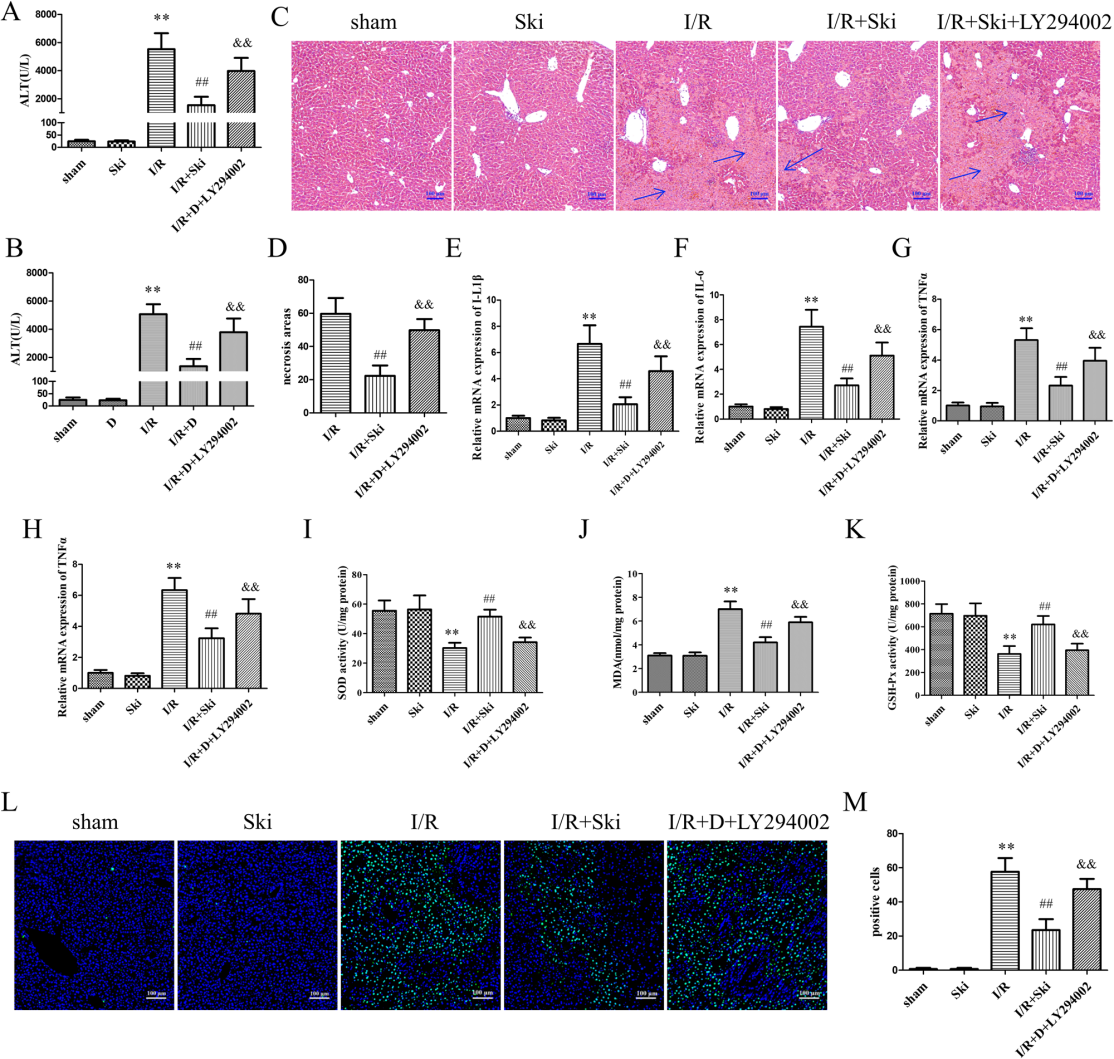
Skimmianine inhibits liver I/R injury via PI3k–AKT pathway. (**A** and **B**) Serum ALS and AST contents of mice in different treatment groups, (**C**) representative H&E staining and (**D**) necrotic area statistical analysis of liver tissue, (**E**–**H**) The mRNA expression of inflammatory cytokines IL-1β, IL-6, TNF-α, and MCP-1 of mice in different treatment groups; (**I**–**K**) MDA content, SOD activity and GSH activity in liver tissues of mice in different treatment groups. (**L**, **M**) TUNEL staining and statistical analysis of liver tissue in different treatment mice. ^**^P < 0.01 versus sham group; ^##^*P* < 0.01 versus I/R group; ^&&^*P* < 0.01 versus I/R + Ski group.

To further verify whether Ski exerts a protective effect against liver I/R via the PI3K–AKT pathway. We used LY294002, an inhibitor of PI3K, to treat mice with intraperitoneal injection. The results showed that LY294002 could well reverse the decrease of ALT and AST levels caused by Ski (*P* < 0.01) (Fig. 7A,B). Also LY294002 could reverse the reduction of liver I/R necrotic area caused by Ski (*P* < 0.01) (Fig. 7C,D). We also confirmed that LY294002 reversed the reduction of inflammation-related indicators IL1-β, IL-6, TNFα and CXCL10 caused by Ski (*P* < 0.01) (Fig. 7E–H). In terms of oxidative stress, LY294002 reversed the reduction of oxidative stress-related indicators MDA, and the elevation of SOD and GSH caused by Ski (*P* < 0.01) (Fig. 7I–K). More importantly, LY294002 reversed the Ski -induced decrease in liver I/R TUNEL positive cells (*P* < 0.01) (Fig. 7L,M). These results could indicate that Ski exerts a protective effect on liver I/R injury via PI3K–AKT pathway.

## Discussion

Cellular inflammatory response, apoptosis, and oxidative stress are important mechanisms of liver I/R injury^38^. Therefore, it is particularly important to find drugs to reduce inflammation, apoptosis, and oxidative stress after liver I/R.

Direct indicators of liver damage are ALT and AST levels in the serum, and the area of liver damage under pathological sections can also be a good indicator of the extent of liver damage^39,40^. In this study, we first demonstrated that Ski was able to attenuate the levels of ALT and AST and necrotic area in mouse liver after I/R injury. These results are direct evidence that Ski attenuates liver I/R injury in mice. Inflammatory injury plays an important role in liver I/R injury, and inflammation-related markers such as IL1-β, IL-6, TNFα, and CXCL10 are often elevated in mouse I/R^41^. MPO has been shown to be a local mediator of inflammation caused by tissue damage and in various inflammatory diseases^42^. Our study confirms that Ski reduces the mRNA expression of IL1-β, IL-6, TNFα, CXCL10 and infiltration of MPO inflammatory cells. These results suggest that Ski exerts an anti-inflammatory effect in liver I/R injury.

Oxidative stress also plays an important function in liver I/R injury. Related indicators such as SOD, GSH-Px, MDA, and DHE staining can detect ROS content^2,38^. We demonstrated in this study that Ski attenuates the production of oxidative stress-related products after liver I/R. This indicates that Ski is able to attenuate liver I/R injury by inhibiting oxidative stress.

Many studies have confirmed the important role of apoptosis in liver injury as well as in liver I/R injury. Targeting the mitigation of apoptosis levels through pharmacological interventions is a promising strategy to cope with liver I/R injury^1,43–45^. Correspondingly, we confirmed the increased apoptosis after I/R injury and cellular H/R model. Also, our results suggest that Ski inhibits the onset of apoptosis in I/R injury. These evidences confirm that Ski can attenuate liver I/R injury by inhibiting apoptosis.

To further investigate the mechanism by which Ski attenuates liver I/R injury. Many studies have confirmed that the PI3K–AKT pathway can play a pivotal role in liver I/R injury^10,22^. Specifically, PI3K–AKT pathway is inhibited in liver I/R injury and activation of PI3K–AKT pathway can reduce liver I/R injury^46,47^. Meanwhile, we learned from research that Ski may interact with PI3K^36^. To further confirm our conjecture, we used molecular docking for validation. Molecular docking is a very effective method for finding drug binding targets. The literature shows that a docking score of less than − 7.0 indicates a strong binding ability of the target to the compound, scores between − 5.0 and − 7.0 indicates a good binding ability of the small molecule to the target, and scores between − 5.0 and − 4.25 indicates a certain binding ability of the compound to the target^48,49^. We docked PI3K with Ski using molecular docking. Ski was able to bind effectively to the active pocket of PI3K with a binding energy of − 6.3 kcal/mol, indicating that Ski is able to bind PI3K more spontaneously. Meanwhile, the WB results further confirmed that Ski could activate the PI3K–AKT pathway.

To further confirm the mechanism of Ski action with hepatic I/R injury. We employed LY294002, a known inhibitor of PI3K phosphorylation, to treat mice. LY294002 could inhibit PI3K phosphorylation and thus act to inactivate PI3K. The results showed that LY294002 could reverse the attenuation of liver I/R injury caused by Ski. These results confirm that Ski exerts a protective effect against liver I/R injury through activation of the PI3K–AKT pathway.

**Figure 8:**
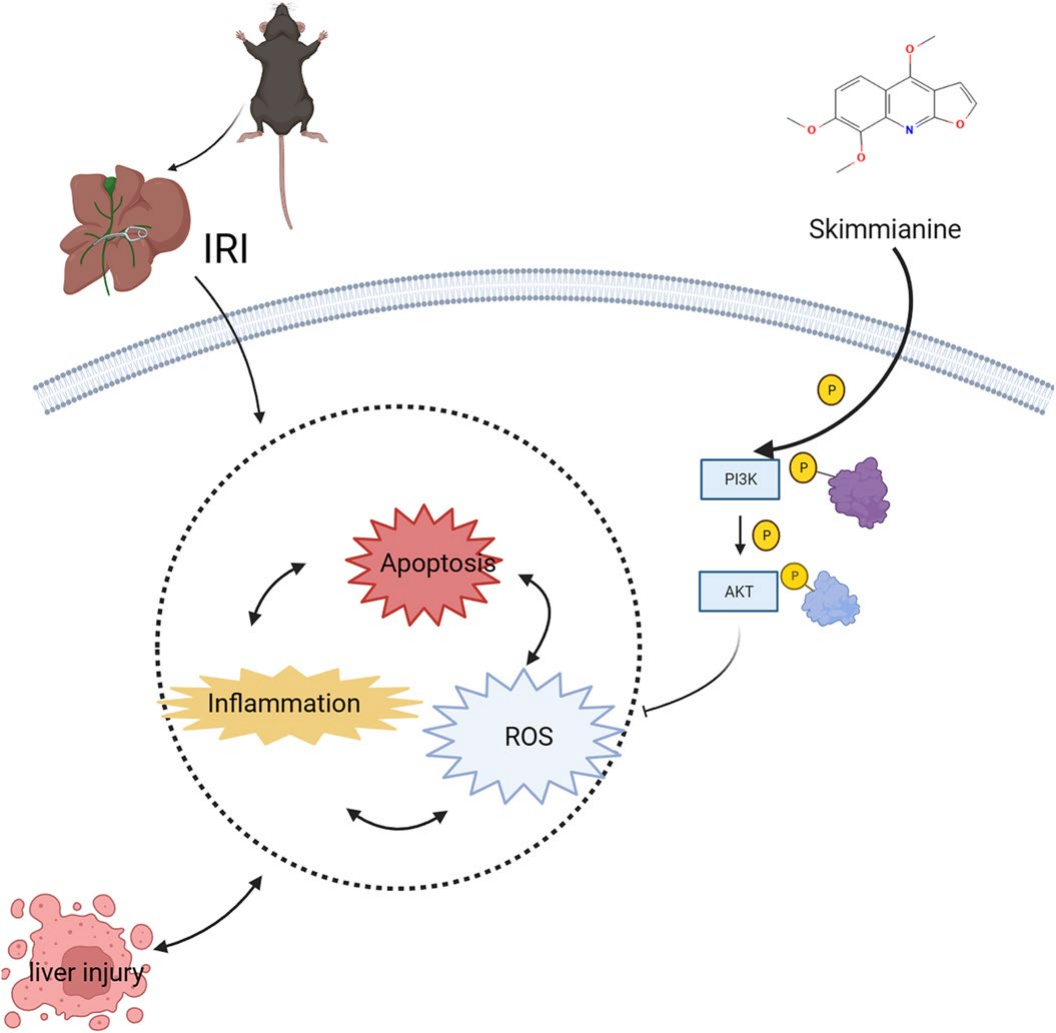
Schematic diagram of the role of Skimmianine in regulating the PI3k–AKT pathway against I/R-induced liver injury.

In conclusion, our study confirm that Ski can alleviate inflammation, apoptosis and oxidative stress after hepatic I/R injury, and that Ski exerts its protective effect on hepatic I/R injury through the PI3K–AKT signaling pathway (Fig. 8). We have made an important contribution by being the first to demonstrate the protective effect of Skimmianine on hepatic I/R injury. This finding opens up a new potential avenue for drug treatment of hepatic I/R injury. Our research provides valuable insights into the therapeutic potential of Ski and highlights its significance as a candidate for further exploration and development in the field of hepatic I/R injury treatment. However, the direct effect of Ski on hepatic I/R injury, and whether Ski can reduce hepatic I/R injury through other pathways needs to be further investigated.

### Supplementary Information

Supplementary Figures.

## Data Availability

The datasets generated during and/or analyzed during the current study are available from the corresponding author on reasonable request.
