# Supplementary material for: Skimmianine attenuates liver ischemia/reperfusion injury by regulating PI3K–AKT signaling pathway-mediated inflammation, apoptosis and oxidative stress
Source: Sci Rep. 2023 Oct 25;13:18232. doi: 10.1038/s41598-023-45354-2 (PMC10600244; doi:10.1038/s41598-023-45354-2)
Supplement: Supplementary file 1 — Supplementary Figures. [file 41598_2023_45354_MOESM1_ESM.pdf]

Figure S1

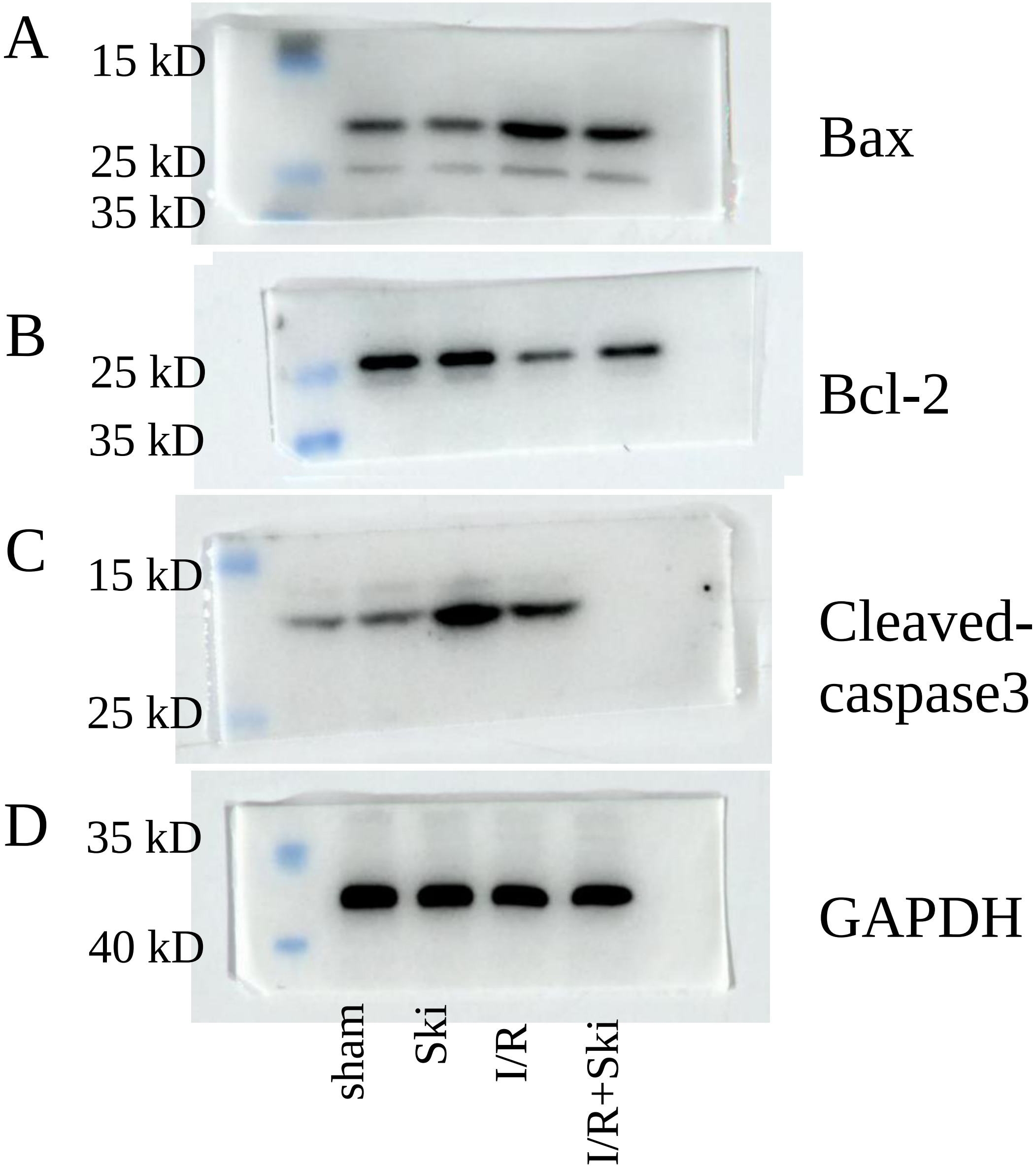

(A) Original western blot band of Bax in figure 4C; (B) Original western blot band of Bcl-2 in figure 4C; (C) Original western blot band of Cleaved-caspase3 in figure 4C; (D) Original western blot band of GAPDH in figure 4C.

Figure S2

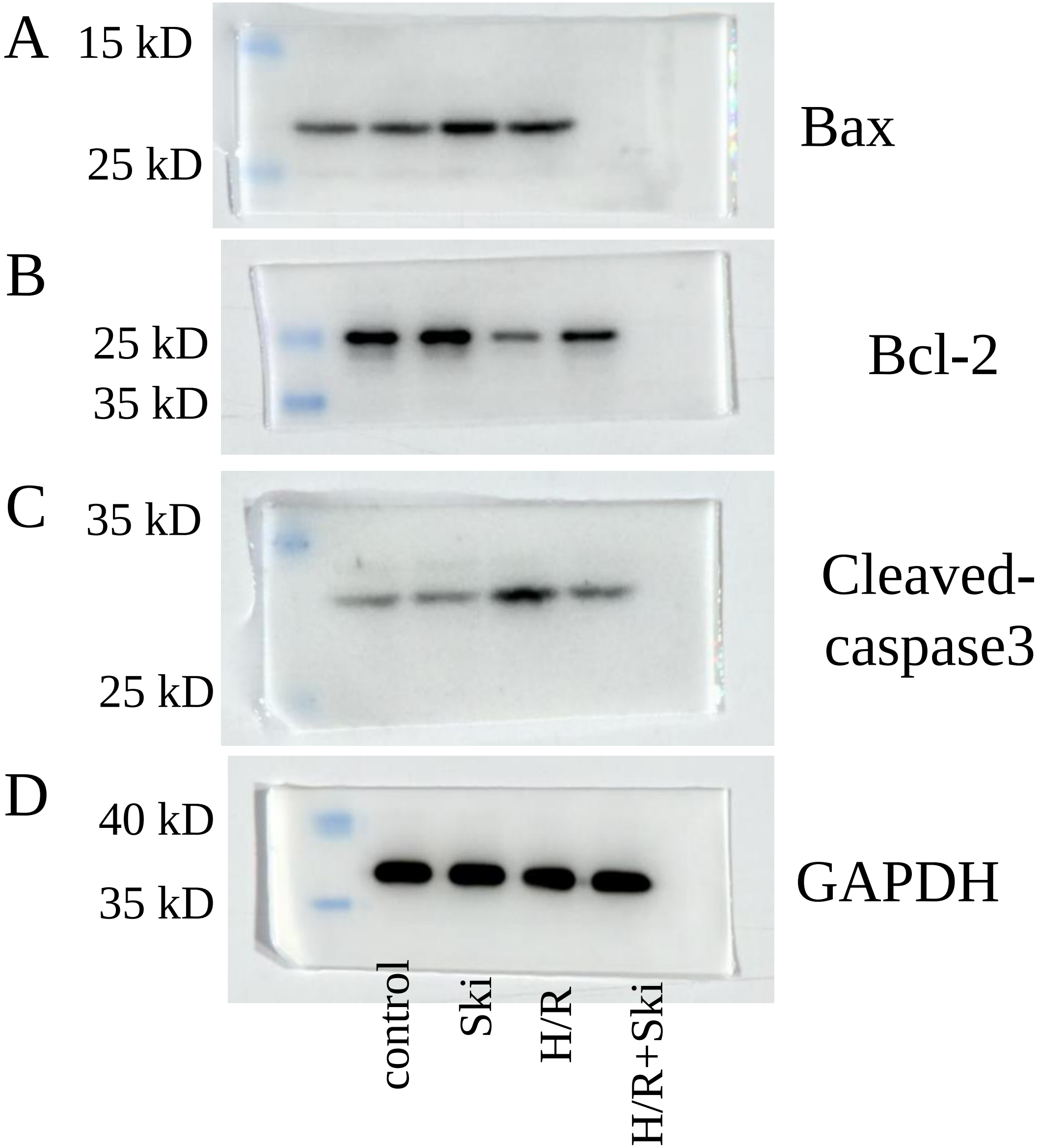

(A) Original western blot band of Bax in figure 5E; (B) Original western blot band of Bcl-2 in figure 5E; (C) Original western blot band of Cleaved-caspase3 in figure 5E; (D) Original western blot band of GAPDH in figure 5E.

Figure S3

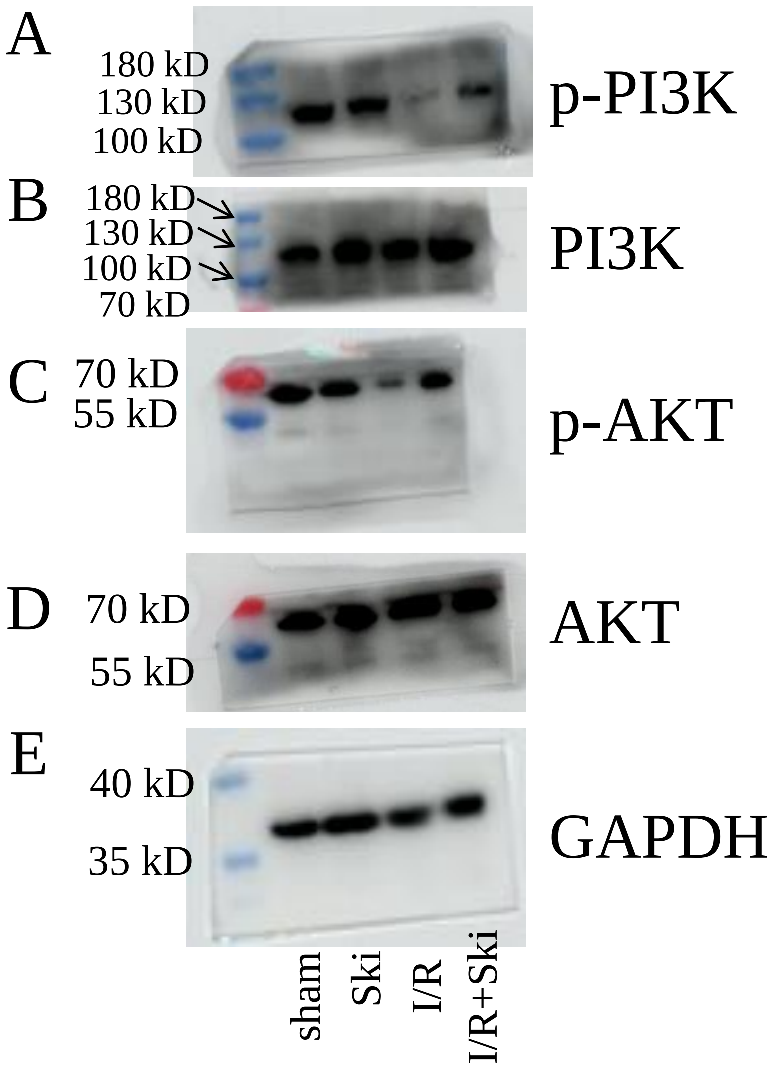

(A) Original western blot band of p-PI3K in figure 6B; (B) Original western blot band of PI3K in figure 6B; (C) Original western blot band of p-AKT in figure 6B; (D) Original western blot band of AKT in figure 6B; (E) Original western blot band of GAPDH in figure 6B.

Figure S4

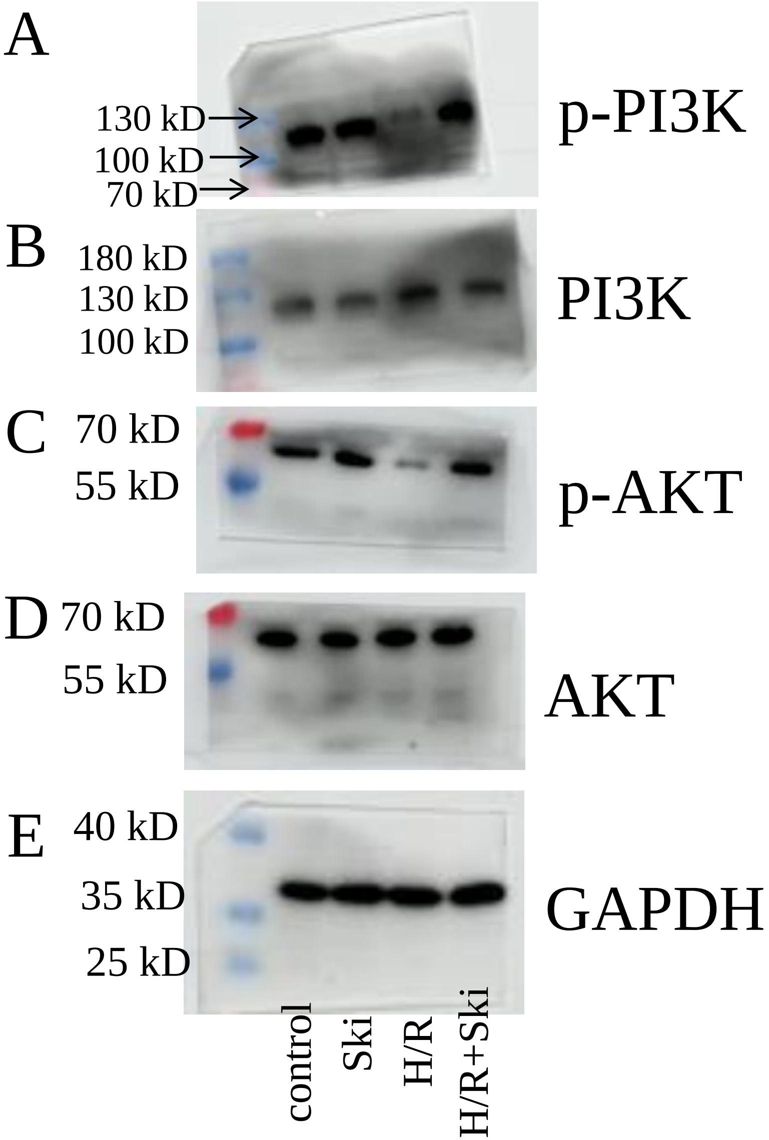

(A) Original western blot band of p-PI3K in figure 6D; (B) Original western blot band of PI3K in figure 6D; (C) Original western blot band of p-AKT in figure 6D; (D) Original western blot band of AKT in figure 6D; (E) Original western blot band of GAPDH in figure 6D.
